# Use of antipsychotic medication and its relationship with bone mineral density: A population-based study of men and women

**DOI:** 10.3389/fpsyt.2022.1004366

**Published:** 2023-01-05

**Authors:** Behnaz Azimi Manavi, Amanda L. Stuart, Julie A. Pasco, Jason M. Hodge, Rasika M. Samarasinghe, D. Kavindi Weerasinghe, Lana J. Williams

**Affiliations:** ^1^School of Medicine, IMPACT—The Institute for Mental and Physical Health and Clinical Translation, Deakin University, Geelong, VIC, Australia; ^2^Barwon Health, Geelong, VIC, Australia; ^3^Department of Medicine-Western Health, The University of Melbourne, St Albans, VIC, Australia; ^4^Department of Epidemiology and Preventive Medicine, Monash University, Melbourne, VIC, Australia; ^5^Geelong Centre for Emerging Infectious Diseases, Geelong, VIC, Australia

**Keywords:** antipsychotic agents, bone density, osteoporosis, absorptiometry (dual-energy X-ray), population-based

## Abstract

**Background:**

Schizophrenia has been shown to be associated with reduced bone mineral density (BMD) and higher fracture risk. However, less is known whether antipsychotic treatment is associated with reduced BMD. Thus, we aimed to examine associations between antipsychotic use and BMD among men and women drawn from the general population.

**Methods:**

This cross-sectional study involved 793 women and 587 men enrolled in the Geelong Osteoporosis Study (GOS). BMD was determined using dual-energy X-ray absorptiometry at the spine and hip. Information regarding socio-economic status (SES), current medication and/or supplementation use, lifestyle factors, and anthropometry was collected. Association between antipsychotic use and BMD was determined using linear regression after adjusting for potential confounders.

**Results:**

Of the group, 33 women (4.2%) and 16 men (2.7%) currently used antipsychotics. Age was identified as an effect modifier in the association between antipsychotic use and BMD for women. Amongst women aged < 60 years, adjusted mean BMD was 11.1% lower at the spine [1.139 (95%CI 1.063–1.216) vs. 1.250 (95%CI 1.223–1.277) g/cm^2^, *p* = 0.005] for antipsychotic users compared to non-users. At the hip, age, weight, and smoking adjusted mean BMD was 9.9% lower [0.893 (95%CI 0.837–0.950) vs. 0.992 (95%CI 0.976–1.007) g/cm^2^, *p* < 0.001] for antipsychotic users in comparison with non-users. The pattern persisted following further adjustments. There was no association detected between antipsychotic use and BMD for women aged 60 years and over and for men.

**Conclusion:**

Our data suggest that antipsychotic medication use is associated with reduced BMD in younger women but not older women or men.

## Introduction

Osteoporosis is a progressive skeletal disease typified by the presence of low bone mineral density (BMD), leading to bone fragility and increased vulnerability to fracture (1). This disease is recognized as a major public health concern worldwide. Between 2017 and 2018, based on self-reported data from the National Health Survey, 924,000 Australians (aged ≥ 44 years) had osteoporosis, accounting for 3.8% of the population. During this time, 93,321 hospitalizations were recorded for minimal trauma fractures among people aged 50 years or older (2). Several recognized osteoporosis risk factors include female sex, advanced age, white race, low body mass index (BMI), smoking, high alcohol intake, glucocorticoid use, sedentary lifestyle, family history, and history of prior fracture (1).

Although not included in the World Health Organization (WHO) list of potential risk factors for osteoporosis, antipsychotic medication use has been previously related to lower BMD (3–11). Antipsychotic medications are a mainstay in the treatment of schizophrenia, with off-label uses for treatment of mood disorders, insomnia, anxiety disorders, agitation, and attention-deficit hyperactivity disorder (ADHD) (12). Antipsychotic use has increased in 10 out of 16 countries, including Australia, between 2005 and 2014 (13). In Australia, 18.5/1,000 of those aged between 20 and 64 and 33.4/1,000 of those aged over 65 years used antipsychotics (13).

While the majority of extant studies investigating the impact of antipsychotics on BMD are in patients with schizophrenia (14–19), other studies have explored the impact of antipsychotics on BMD in other patients groups such as those with bipolar disorder (18, 20) and autism (21). Consequently, confounding by indication is possible. Although, a review of available literature between 1966 and 2010 by Crews and Howes (11) concluded that patients taking antipsychotic medication, regardless of diagnosis, showed lower BMD compared to controls (11). Antipsychotic medication has also been associated with a greater likelihood of falls and osteoporotic fracture (22).

Given the recent increase in usage of antipsychotics and morbidity and mortality associated with osteoporotic fracture and the issue of confounding by indication, we aimed to determine whether a relationship between antipsychotic medication use and BMD exists in a population-based sample of adults.

## Materials and methods

### Study design and subjects

Data for this cross-sectional study were drawn from the Geelong Osteoporosis Study (GOS). GOS is an on-going, population-based cohort study of adults randomly selected from the electoral roll for the Barwon Statistical Division (south-eastern Australia) (23).

At baseline (between 1994 and 1997), 1,494 women aged 20–94 years (median age 54.2 years; response rate 77.1%) were recruited and have returned for assessment 2-, 4-, 6-, 8-, 10-, and 15-years post baseline. In 2005, a further 246 women aged between 20 and 29 years were recruited using the same sampling method to supplement the full adult age range for women samples. Between 2001 and 2006, 1,540 men aged 20–97 years (median age 56.0 years; response rate 67.0%) were recruited and returned for assessment 5- and 15-years post baseline. Extensive medical, lifestyle, socio-demographic and clinical data have been collected at each follow-up (23).

For the present study, antipsychotic users were drawn from across the GOS study period (1994–2019). Participants who reported using antipsychotic medication at any assessment were included as an “antipsychotic user” (*n* = 49; 32.6% men) and “antipsychotic non-users” were drawn from the most recent follow-up (*n* = 1,331; 42.9% men).

This study was approved by Barwon Health’s Human Research Ethics Committee (ID 92/01 and 00/56). All participants provided informed and written consent for using their data.

### Assessments

#### Outcome

Areal BMD (g/cm^2^) was assessed at the spine (L2–L4, posterior–anterior projection) and femoral neck using dual-energy X-ray absorptiometry. Women were scanned on Lunar DPX-L (software version 1.31; Lunar, Madison, WI, USA) as were the first 554 men until it was outmoded and replaced with a GE-Prodigy (Prodigy; GE Lunar, Madison, WI, USA) (23). Technicians completed daily calibrations of the densitometer with equipment-specific phantoms.

#### Exposures

Current medication use and duration were documented at each assessment, with participants asked to bring their medication containers for accurate recording. Use of antipsychotics and medications known to affect bone including hormone therapy, thyroid medication, bisphosphonates, oral glucocorticoids, and calcium/vitamin D supplements were included in this study.

Height was measured to the nearest 0.1 cm with a wall-mounted Harpenden stadiometer and weight to the nearest 0.1 kg with an electronic scale. Alcohol intake was determined by a validated food frequency questionnaire and recorded in grams per day (24). Current smoking was self-reported based on the use of manufactured or hand-rolled cigarettes, cigars, or pipes and grouped as “current smoker” or “non-smoker.” Habitual physical activity was documented on a seven-point scale from “very active” (moves, walks, and works energetically) through to “bedfast” (not able to walk) based on Metabolic Equivalent of Task Values, as previously described (25) and dichotomized as active vs. inactive. Socio-economic status (SES) was determined by Socio-Economic Index for Areas (SEIFA) index scores of the residential address of participants based on the 2006 Australian Bureau of Statistics census data. SEIFA scores were used to determine an Index of Relative Socio-economic Advantage and Disadvantage (IRSAD) accounting for income ranging low to high, and type of occupation from unskilled employment to professional positions. Scores range from 1 (most disadvantaged) to 5 (most advantaged) (26).

### Statistical analyses

Minitab (version 18; Minitab, State College, Pennsylvania) and SPSS (version 28) were used for statistical analyses. Differences in characteristics between antipsychotic users and non-users were presented separately for women and men. Continuous parametric and non-parametric variables were analyzed using *t*-tests and Kruskal–Wallis, respectively. Discrete variables were analyzed using chi-square tests or Fisher’s exact tests when expected cell counts were less than five.

The association between antipsychotic use (exposure of interest) and BMD (outcome) was explored using multiple linear regression. In backward stepwise regression, covariates including age, weight, height, alcohol intake (g/d), current smoking status, habitual physical activity, SES, and medications known to influence bone were tested, with significant variables retained, resulting in best models. Models were developed separately for women and men. Interactions between covariates were tested in the final models. *p*-values < 0.05 were accepted as significant.

The female sample (antipsychotic user = 33, non-user = 760) had 98% power to detect small effect size, and the male sample (antipsychotic user = 16, non-user = 571) had 93% power to detect small effect size (27).

## Results

### Women

Thirty-three women reported using antipsychotic medication; 7 (21.2%) used haloperidol, 7 (21.2%) olanzapine, 7 (21.2%) quetiapine, 7 (21.2%) trifluoperazine, 2 (6.1%) chlorpromazine, 1 (3.0%) aripiprazole, 1 (3.0%) risperidone, and 1 (3.0%) thioridazine. Median duration of antipsychotic use was 42 months (IQR 18–142). Antipsychotic users were more likely to use antidepressant medication and were less active; otherwise, there were no differences between the groups with regard to age, weight, height, current smoking status, alcohol consumption, SES, and use of medications known to affect bone (Table 1).

**TABLE 1:** Characteristics of antipsychotic users and non-users according to age for women.

	All			23–59 years			60–95 years		
	Antipsychotic user	Non-user	*p*	Antipsychotic user	Non-user	*p*	Antipsychotic user	Non-user	*p*
									
	*n* = 33	*n* = 760		*n* = 17	*n* = 439		*n* = 16	*n* = 321	
Age (year)	58.4 (43.5–70.1)	56.0 (43.2–68.0)	0.72	44.0 (34.0–51.5)	45.5 (37.0–52.5)	0.46	70.1 (65.6–76.3)	70.6 (65.0–76.3)	0.86
Weight (kg)	77.3 (56.4–95.5)	71.5 (62.3–84.0)	0.65	80.1 (59.7–96.5)	71.5 (62.1–84.2)	0.40	74.8 (53.8–92.9)	71.6 (62.6–83.1)	0.82
Height (m)	1.6 ± 0.1	1.6 ± 0.1	0.55	1.6 ± 0.1	1.6 ± 0.1	0.05	1.6 ± 0.1	1.6 ± 0.1	0.36
Current smoking (current)	7 (21.2%)	84 (11.2%)	0.08	6 (35.3%)	65 (15.1%)	0.02	1 (6.2%)	19 (5.9%)	1
Habitual physical activity (active)	13 (39.4%)	547 (73.5%)	<0.001	9 (52.9%)	364 (84.8%)	<0.001	4 (25.0%)	183 (58.1%)	<0.001
Alcohol intake (g/d)	0.0 (0.0–5.7)	1.43 (0.0–8.6)	0.06	0.0 (0.0–28.6)	2.8 (0.0–7.9)	0.89	0.0 (0.0– 0.0)	1.4 (0.0–8.6)	0.02
Socioeconomic status (current)			0.37			0.82			0.27
Quintile 1 (most disadvantaged)	3 (12.0%)	119 (15.7%)		1 (7.1%)	60 (13.7%)		2 (18.2%)	59 (18.4%)	
Quintile 2	5 (20.0%)	79 (10.4%)		2 (14.3%)	43 (9.8%)		3 (27.3%)	36 (11.2%)	
Quintile 3	7 (28.0%)	294 (38.7%)		4 (28.6%)	168 (38.3%)		3 (27.3%)	126 (39.2%)	
Quintile 4	7 (28.0%)	145 (19.1%)		4 (28.5%)	93 (21.2%)		3 (27.3%)	52 (16.2%)	
Quintile 5	3 (12.0%)	123 (16.2%)		3 (21.4%)	75 (17.1%)		0 (0.0%)	48 (14.9%)	
Medication use (current)									
Antidepressant	16 (48.5%)	128 (16.9%)	<0.001	10 (58.8%)	61 (13.9%)	<0.001	6 (37.5%)	67 (20.9%)	0.11
Hormone therapy	2 (6.1%)	27 (3.5%)	0.28	0 (0.0%)	16 (3.6%)	1	2 (12.5%)	11 (3.4%)	0.12
Thyroid agents	1 (3.0%)	23 (3.3%)	1	0 (0.0%)	26 (5.9%)	0.61	1 (6.2%)	27 (8.4%)	0.76
Calcium and/or vitamin D	9 (27.3%)	166 (21.8%)	0.46	4 (23.5%)	63 (14.3%)	0.29	103 (32.1%)	5 (31.2%)	0.94
Bone mineral density (g/cm^2^)									
PA-spine	1.184 ± 0.224	1.203 ± 0.184	0.63	1.164 ± 0.210	1.247 ± 0.166	0.13	1.206 ± 0.242	1.143 ± 0.192	0.32
Femoral neck	0.910 ± 0.152	0.923 ± 0.145	0.63	0.913 ± 0.145	0.978 ± 0.135	0.08	0.906 ± 0.165	0.844 ± 0.123	0.18

Values are given as median (IQR), mean (± SD) or frequency (%).

Age was identified as an effect modifier in the association between antipsychotic use and BMD, with the relationship differing for those aged < 60 years and > 60 years. Among younger women (< 60 years; *n* = 456), age, weight, calcium/vitamin D supplement, and smoking adjusted mean BMD was 11.1% lower at the spine for antipsychotic users compared to non-users [1.139 (95%CI 1.063–1.216) vs. 1.250 (95%CI 1.223–1.277) g/cm^2^, *p* = 0.005]. At the hip, age, weight, and smoking adjusted mean BMD was 9.9% lower for antipsychotic users compared to non-users [0.893 (95%CI 0.837–0.950) vs. 0.992 (95%CI 0.976–1.007) g/cm^2^, *p* < 0.001]. Alcohol consumption, height, activity level, SES, and medications known to affect bone did not contribute to the models. There was no significant association observed between antipsychotic use and BMD at either the spine or hip for women aged 60 years or older (both *p* > 0.05) (Table 2).

**TABLE 2:** Unadjusted and best models showing associations between antipsychotic use and BMD for women and men.

	Model I: Unadjusted			Model II: Best model*		
	β	*SE*	*p*	β	*SE*	*p*
**Women**						
**Spine^¥^**						
<60	–0.083	0.041	0.046	–0.111	0.039	0.005
≥60	0.063	0.050	0.207	0.036	0.045	0.427
**Hip^£^**						
<60	–0.065	0.033	0.051	–0.099	0.029	<0.001
≥60	0.062	0.034	0.069	0.051	0.029	0.076

**Men^§^**						
Spine	–0.042	0.050	0.399	–0.018	0.046	0.692
Hip	0.040	0.035	0.251	–0.003	0.030	0.911

*Best models adjusted for: ^¥^age, weight, calcium/vitamin D, and smoking, ^£^age, weight, and smoking, ^§^age, weight, calcium/vitamin D, and bisphosphonate. β, regression coefficient; SE, standard error.

### Men

At the time of assessment, 16 men (2.7%) reported using antipsychotic medication; 7 (43.7%) used olanzapine, 4 (25.0%) quetiapine, 1 (6.2%) aripiprazole, 1 (6.2%) clozapine, 1 (6.2%) paliperidone, 1 (6.2%) risperidone, and 1 (6.2%) used trifluoperazine. Median duration of use was 28 months (IQR 10–71). Antipsychotic users were younger, consumed less alcohol and were more likely to smoke and use antidepressants; there were no differences in weight, height, activity, SES, and use of medications known to affect bone between the users and non-users (Table 3).

**TABLE 3:** Characteristics of antipsychotic users and non-users for men.

	Antipsychotic user	Non-user	*p*
	*n* = 16	*n* = 571	
Age (year)	52.6 (38.8–65.8)	64.3 (52.5–73.6)	0.03
Weight (kg)	89.8 (75.4–99.4)	83.0 (75.1–92.9)	0.18
Height (m)	1.7 ± 0.1	1.7 ± 0.1	0.41
Current smoking (current)	6 (37.5%)	47 (8.25%)	<0.001
Habitual physical activity (active)	9 (56.2%)	428 (75.1%)	0.08
Alcohol intake (g/d)	0.4 (0.0–11.6)	10.7 (2.5–27.3)	<0.001
Socioeconomic status (current)			0.51
Quintile 1 (most disadvantaged)	4 (30.8%)	91 (16.16%)	
Quintile 2	3 (23.1%)	118 (21.0%)	
Quintile 3	1 (7.7%)	128 (22.7%)	
Quintile 4	4 (30.8%)	152 (27.0%)	
Quintile 5	1 (7.7%)	74 (13.1%)	
Medication use (current)			
Antidepressant	7 (43.7%)	57 (10.0%)	<0.001
Hormone therapy	0 (0.0%)	10 (1.7%)	1
Thyroid agents	0 (0.0%)	14 (2.4%)	1
Calcium and/or vitamin D	4 (25.0%)	66 (11.5%)	0.10
Bone mineral density (g/cm^2^)			
AP-spine	1.280 ± 0.130	1.320 ± 0.200	0.45
Femoral neck	1.010 ± 0.145	0.966 ± 0.133	0.30

Values are given as median (IQR), mean (± SD) or frequency (%).

Before and after adjustment for age and weight, there was no relationship detected between antipsychotic use and BMD at the spine or hip in men (all *p* > 0.05) (Table 2).

## Discussion

In this population-based study, antipsychotics use was associated with lower BMD at the spine and hip for women aged under 60 years compared to non-users. These relationships were sustained following further adjustment for lifestyle factors and medications known to affect bone. This pattern was not observed among older women or men.

The association between antipsychotic medication and low BMD is well documented in clinical samples (10), however, there are few population-based studies. In a large, population-based study of 6,820 females (aged ≥ 50 years) with osteoporosis (cases) and 20,247 age-, sex- and ethnicity-matched controls, 88 cases (1.3%) and 124 controls (0.6%) used atypical antipsychotics (*p* < 0.0001). Following adjustments, use of atypical antipsychotics was associated with a greater risk of osteoporosis (adjusted odds ratios 1.55, 95%CI 1.06–2.28), with risk being independent of diagnoses of a mental disorder (28). In another large study of 68,730 individuals (9.4% male) aged 40 years and older by Bolton et al., antipsychotic use was associated with higher risk of any incident major osteoporotic fracture (HR 1.43; 95%CI, 1.15–1.77; *p* < 0.05) as well as incident hip fracture (HR 2.14; 95%CI, 1.52–3.02; *p* < 0.05) independent of diagnoses of a mental disorder (29).

Essentially, typical antipsychotics prevent dopamine signaling, while atypical antipsychotics also avoid signaling serotonin (30). Atypical antipsychotics are known to have broader effectiveness, however, more metabolic adverse effects compared to first generation (30). Although not possible in the present study due to power constraints, others have investigated differences between typical and atypical antipsychotics in their effect on bone. Bolton et al. (28) found women aged over 50 years using atypical antipsychotics had a 1.5–fold higher risk of osteoporosis compared to controls, however, this pattern was not observed for typical antipsychotics. A later study by Wang et al. (17), provided contradictory results, reporting a reduction in BMD and increased risk of osteoporosis for typical antipsychotic users but not atypical antipsychotic users, compared to controls, following 12 months of treatment (17). Some have further investigated duration of use, finding chronic use to be related with reduced bone mineralization (3, 7, 8, 31). A more recent study exploring BMD in patients on antipsychotics found a negative association between duration of antipsychotic use and BMD at the femoral neck (4).

In the current study, age was found to be an effect modifier in the relationship between antipsychotic use and BMD in women, in that the younger women (< 60 years) taking antipsychotics had lower BMD in comparison with non-users and could be considered as being at higher risk of developing osteoporosis. There are several possible reasons for this relationship. It is estimated that peak bone mass is achieved in an individual around 30 years of age (32), with BMD decreasing thereafter (33). Since the onset of psychiatric disorders, particularly schizophrenia, often occurs during adolescence and young adulthood, as with the commencement of antipsychotic medication and poor lifestyle choices such as cigarette smoking, it is possible peak bone mass is affected (9). It has also been suggested that tolerance to the effects of antipsychotic medications may develop as individuals age, or that young women are more sensitive to the prolactin-elevating effects of the medications (9).

Poor bone quality observed in individuals with schizophrenia and other psychiatric disorders (34) has previously been attributed to certain lifestyle factors including cigarette smoking, poor dietary intake, alcohol abuse, lack of exercise, and low vitamin D (33). It has been previously reported that people taking antipsychotics who do not smoke and exercise on a regular basis have higher lumbar spine BMD compared to those who smoke and do not exercise regularly (4). It is reported women with psychotic disorders taking antipsychotic medications have some risk factors for low BMD including low BMI, prolactin level, low vitamin D, and serotonergic antidepressant use (16). Lifestyle factors including alcohol intake, smoking, activity level, and medications known to affect bone were taken into consideration in this paper but did not explain the observed relationship among the younger women.

A major strength of this study is the large population-based sample of men and women that were not selected on the basis of disease status. Confounding by indication is frequently encountered in observational studies investigating medication effects. A novel aspect of this study was our ability to determine whether an association prevailed between antipsychotic use and BMD, independent of schizophrenia, which has been previously shown to be associated with poor bone health. Furthermore, the sample spanned the full adult age range, and a large range of potential cofounders were considered in the statistical models. We recognize that this study has some limitations, including the number of antipsychotic users which could be due to a “healthy participant bias.” Further investigation of dose and duration of antipsychotic use was not possible. The cross-sectional design of this study prevented conclusions on bone loss and specific confounders operative over the time period. Finally, prolactin levels and markers of reproductive function were not measured, thus we were unable to discuss the impact of hormonal changes related to antipsychotic use on bone. Studies concentrating on the impact of different antipsychotic agent use on BMD in a large population-based samples are needed.

## Conclusion

This study demonstrated that antipsychotic use is associated with lower BMD in women aged under 60 years but not in older women or men. Monitoring of bone health in this subpopulation may be warranted, with future research into underlying mechanisms needed.

## Data availability statement

The raw data supporting the conclusions of this article will be made available by the authors, without undue reservation.

## Ethics statement

The studies involving human participants were reviewed and approved by the Barwon Health’s Human Research Ethics Committee (ID 92/01 and 00/56). The patients/participants provided their written informed consent to participate in this study.

## Author contributions

BAM, JP, and LW contributed to the study conception and design of this study. BAM, AS, JP, and LW analyzed and interpreted the data. BAM and LW drafted this manuscript. All authors reviewed, edited, and approved the final version.
